# Realizing Tunable Inverse and Normal Doppler Shifts in Reconfigurable RF Metamaterials

**DOI:** 10.1038/srep11659

**Published:** 2015-06-26

**Authors:** Jia Ran, Yewen Zhang, Xiaodong Chen, Kai Fang, Junfei Zhao, Yong Sun, Hong Chen

**Affiliations:** 1 Tongji University, Shanghai, 200092, China; 2 University of Electronic Science and Technology of China, Chengdu, 610054, China; 3 Queen Mary University of London, E1 4NS, UK

## Abstract

The Doppler effect has well-established applications in astronomy, medicine, radar and metrology. Recently, a number of experimental demonstrations of the inverse Doppler effect have begun to appear. However, the inverse Doppler effect has never been observed on an electronically reconfigurable system with an external electromagnetic wave source at radio frequencies (RF) in experiment. Here we demonstrate an experimental observation of the inverse Doppler shift on an electronically reconfigurable RF metamaterial structure, which can exhibit anomalous dispersion, normal dispersion or a stop band, depending on an applied bias voltage. Either inverse or normal Doppler shift is realized by injecting an external RF signal into the electronically reconfigurable metamaterial, on which an electronically controllable moving reflective boundary is formed. The effective velocity of this boundary and the resulting frequency shift can be tuned over a wide range by a digital switching circuit. This work is expected to open up possibilities in applying the inverse Doppler effect in wireless communications, radar and satellite navigation.

The Doppler effect is caused by the fact that the relative motion between a source and a detector will lead to a frequency shift in the received signal. In media of normal dispersion (right-handed), an approaching source increases the received signal frequency, whereas a receding source decreases it. However, in media of anomalous dispersion (left-handed) it has been proved in theory that an inverse Doppler effect occurs, that is, an approaching source decreases the received signal frequency, whereas a receding source increases it^1^. This counter-intuitive inverse Doppler effect was theoretically investigated by Veselago in 1968 in a material with simultaneously negative permittivity and negative permeability, since the group velocity and phase velocity are in the opposite directions in these double negative materials (DNG)^1^. Such a material, now known as a metamaterial, was realized physically by Pendry, Smith *et al*. around 2000^2^,^3^,^4^ and has since been extensively studied by others^5^,^6^,^7^,^8^,^9^,^10^.

Until now there have been only a few inferred measurements of the inverse Doppler effect in metamaterials at radio frequencies. Based on the approach proposed by Kozyrev *et al*.^11^,^12^, the first experimental demonstration of the inverse Doppler effect at radio frequency was reported by Seddon and Bearpark^13^. In their experiment, the RF waves were not injected directly from an external source, but were excited by a moving shock wave on the magnetic nonlinear transmission line (an internal source) and bounced between the front of the pump pulse and the input terminal of the line. Hence, their work, referred to as the indirect experimental measurement in Ref. 14, not only causes a controversy in the interpretation of the results^15^,^16^, but also is not complete, since the frequency down-shifted signal does not exist^17^. Leong *et al*.^18^ and Yuan *et al*.^19^ have also demonstrated the inverse Doppler effect along a left-handed transmission line using an internal source in the circuit simulation. Their internal effective moving source was modeled by using sequential fast switches on the transmission line. Also, Kozyrev *et al*.^20^ observed the inverse Doppler shift with an external RF signal on a magnetic nonlinear transmission line in simulation lately. In addition, the inverse Doppler effect was observed experimentally at optical and acoustic frequencies^14^,^21^, and was also observed when magnons (magnetostatic spin wave ) reflected from a moving solid object and magnonic crystal in the gigahertz frequency range^22^,^23^.

This has motivated us to explore an experimental observation of the inverse Doppler effect with an external RF source on an electronically reconfigurable composite right-left handed transmission line (CRLH TL), which was originally proposed by Eleftheriades and Carloz *et al*. as a convenient 1-D metamaterial structure^24^,^25^,^26^,^27^. By loading such a transmission line with the varactors^28^, we have further extended its reconfigurability, by arranging that the dispersion characteristics of each line section can be controlled electronically through a digital switching circuit (later referred to as a reflective boundary controller). By controlling the bias voltage, each section of the transmission line can exhibit normal dispersion (right-handed), anomalous dispersion (left-handed/DNG), or a stop band (band gap). An electronically controllable moving reflective boundary can be formed between the regions of either normal transmission, anomalous dispersion and a band gap, by means of the reflective boundary controller. When an external RF signal is injected into this tunable transmission line, shown schematically in Fig. 1a, the reflected wave from the moving reflective boundary can be treated as an effective source approaching or receding from the detector. While the passband section of the transmission line is set to be in normal dispersion mode (right-handed) by choice of its bias voltage, the group and phase velocities of the wave are in the same direction. Hence, the normal Doppler effect occurs in this situation. If the passband section is set to be in anomalous dispersion mode (left-handed), the group and phase velocities of the wave are in opposite directions. Consequently, when the reflective boundary is approaching or receding from the detector, the phase velocity of the wave is actually receding from or approaching the detector, leading to the inverse Doppler effect. Hence, either the inverse Doppler effect or the normal Doppler effect (both frequency up-shifted and down-shifted) can be realized by applying a suitable switching mode of the controlling voltage on this CRLH transmission line.

## Results

### Theoretical Doppler frequency shifts

The frequency of the reflected wave *f*_*r*_ and the frequency of the incident wave *f*_*i*_ have following relationship^13^:
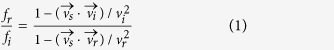
where *v*_*s*_, *v*_*i*_, *v*_*r*_ are the velocities of the moving reflective boundary, incident wave phase velocity and reflected wave phase velocity, respectively. Therefore the Doppler shift can be calculated as:
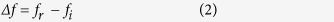


Equations (1) and (2) have been used to predict the inverse and normal Doppler shifts. The difference between the inverse and normal Doppler effects is that the directions of the electromagnetic waves are opposite, since the phase velocity is antiparallel with the group velocity in double negative materials and parallel in normal materials. The dispersion of the CRLH TL is linear and the velocity of the incident wave varies slightly in the Doppler shift range, as shown in Supplementary Fig. 6, hence we have approximately 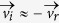. The theoretical velocity of the incident wave in the transmission line can be obtained easily from the theoretical dispersion function (see Supplementary Information S-II for details). The Doppler frequency shifts have also been obtained by using Keysight (Agilent) ADS (Advanced Design System) simulator.

### Characteristics of the tunable composite right-left handed transmission line

**Figure 1:**
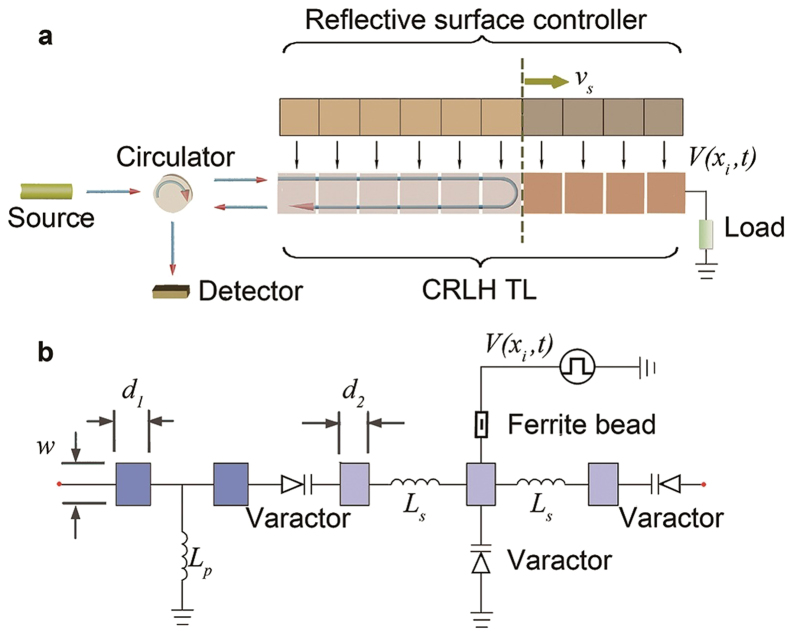
Illustration of the experimental set-up used to demonstrate inverse and normal Doppler shifts. **a**, The whole set-up used to test Doppler shifts. *V*(*x*_*i*_,*t*)(*i* = 1,2…14) are the bias voltages provided to the CRLH TL units. Two different colors on the reflective boundary controller stand for two different voltages, which leads to the different characteristics of the corresponding units of the transmission line. The units of the transmission line in gray lie in passband (either right-handed or left-handed), while the CRLH TL units in brown are set in band gap. The reflective boundary at the interface between the two kinds of units can move at the velocity of *v*_*s*_ by switching the bias voltages supplied by the external controlling circuit. **b**, Schematic of the unit of the CRLH TL. The reflective boundary controller provides the bias voltage *V*(*x*_*i*_,*t*) to the varactors through a ferrite bead which prevents the incident wave propagating from the CRLH TL to the controller.

The unit of the composite right-left handed transmission line comprised 5 sections of microstrip line, each loaded with two NXP BB135 varactor diodes in series and one in parallel, as shown in Fig. 1b. In addition, two inductors *L*_*S*_ in series and one *L*_*P*_ in parallel are chosen to select the operating frequency. When the bias voltages changes, the capacitances of the three varactors change - leading to variation of the dispersion curve. The characteristics of the CRLH TL unit are simulated using Keysight (Agilent) ADS simulator, a commonly used RF circuit modeling tool.

**Figure 2:**
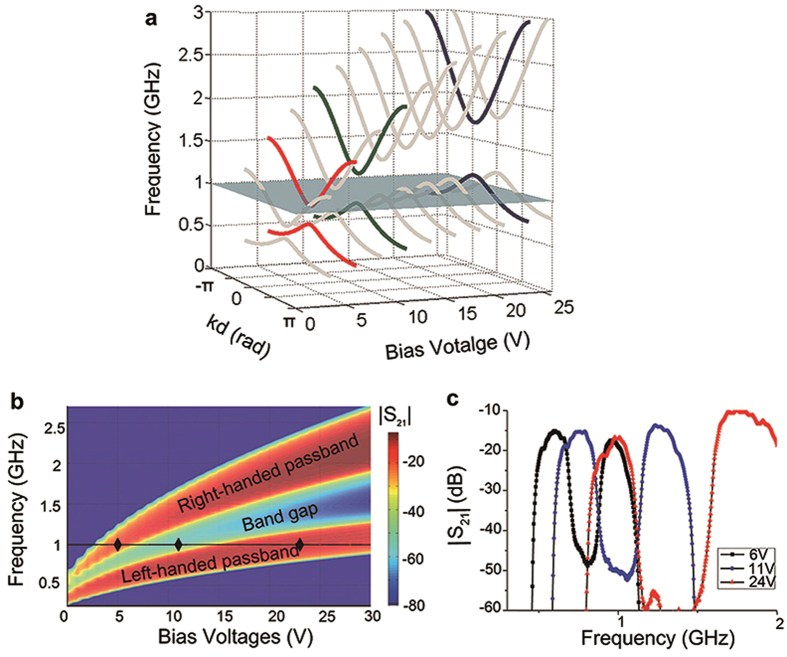
The voltage-dependent transmission characteristics. **a**, Theoretical dispersion curves under different bias voltages. The gray plane indicates the working frequency 1 GHz, while the red, green and dark curves correspond to three typical bias voltages (6 V, 11 V and 24 V). **b**, Simulated |*S*_21_| under different bias voltages. The branch above is the right-handed passband while the branch blow is the left-handed passband. The dark diamond markers are a set of bias voltages (5 V, 11 V and 23 V) at 1 GHz for achieving the equivalent transmission characteristics in **a**. **c**, Measured |*S*_21_| verse frequency at the three bias voltages shown in a.

Figure 2a shows the theoretical dispersion curves of the CRLH TL as a function of the bias voltages which are calculated by considering the transmission line as a cascade network (see Supplementary Information S-II). The gray plane at 1 GHz cuts across the upper branches (right-handed passband), the band gap, and the lower branches (left-handed passband) of the dispersion curves under different bias voltages. The red, green and dark curves correspond to the dispersion under bias voltages of 6 V, 11 V and 24 V, respectively. Figure 2b shows the simulated transmission, in terms of |*S*_21_|, under different bias voltages. Because of inaccuracy of the varactor circuit model in ADS, the bias voltages in simulation needed to be set slight differently from those in the theoretical model, i.e. 5 V, 11 V and 23 V for achieving the equivalent transmission characteristics (as shown in Supplementary Fig. 7b). Both figures indicate that with the increased bias voltages, the propagation characteristics at 1 GHz change from right-handed transmission to a band gap (stop-band transmission), and then to left-handed transmission. The measured transmission |*S*_21_| at 1 GHz at three voltages of 6 V, 11 V and 24 V is illustrated in Fig. 2c.

In the experiments, a 14-unit CRLH transmission line was built and connected to a reflective boundary controller that separately provides the bias voltages to each transmission line unit, as shown in Fig. 1a. If the left-hand sections of the transmission units are set in the passband mode and the right-hand sections are set in the band gap mode by providing different bias voltage to the transmission line units, a reflective boundary will appear at the interface between the passband and the band gap sections.

The reflective boundary can move either towards or away from the detector by regulating the bias voltages to each CRLH TL unit and by choosing the proper wave incident direction. For convenience of testing, the wave is emitted into the CRLH TL from either the left end or the right end. For instance, as Fig. 1a shows, bias voltages of the left CRLH TL units (gray) are at either 6 V or 24 V, setting these units in the transmission mode (right-handed or left-handed), while the bias voltages of right CRLH TL units (brown) are at 11 V, setting these units in band gap mode. By this means a reflective boundary is formed at the interface of these two groups of CRLH TL units. Changing the bias voltages of the TL unit to the right of the reflective boundary would switch the unit from band gap to passband, so the reflective boundary will move one unit to the right. By stepping the change in bias voltages sequentially rightwards, we obtain a rightwards moving reflective boundary, moving away from the detector at the left hand end of the structure. The effective velocity of the boundary *v*_*s*_ can be controlled by regulating the time interval of the pulse signal in the reflective boundary controller and is positive when the boundary moves away from the source. The reflective boundary could be considered moving smoothly when the wavelength is considerably larger than the length of the transmission unit. For an incident wave in the leftwards direction, we can achieve a reflective boundary moving towards the detector if the left part of the TL is set in band gap mode while the right part is set in passband mode. By these means both inverse and normal Doppler shifts can be realized in the experiments under proper control of the bias voltages and the location of the incident wave.

### Measured Doppler frequency shifts

In the experiments, the wavelength of the incident wave at 1 GHz is 88.6 mm in the left-handed passband and 151.1 mm in the right-handed passband; both are much longer than each unit of the CRLH TL (*d*_0_ = 14.2 *mm*). The effective velocity of the reflective boundary is in the range 17.8–71.0 km/s. The corresponding inverse Doppler shift is between –0.385 MHz and –1.465 MHz (for *v*_*s*_ < 0), between +1.420 MHz and +0.325 MHz (for *v*_*s*_ > 0) and the normal Doppler shift is between +1.125 MHz and +0.26 MHz (for *v*_*s*_ < 0), or between –0.25 MHz and –1.095 MHz (for *v*_*s*_ > 0). The direction of the incident wave should be different according to the different sets of edges and bias voltages, as discussed below.

**Figure 3:**
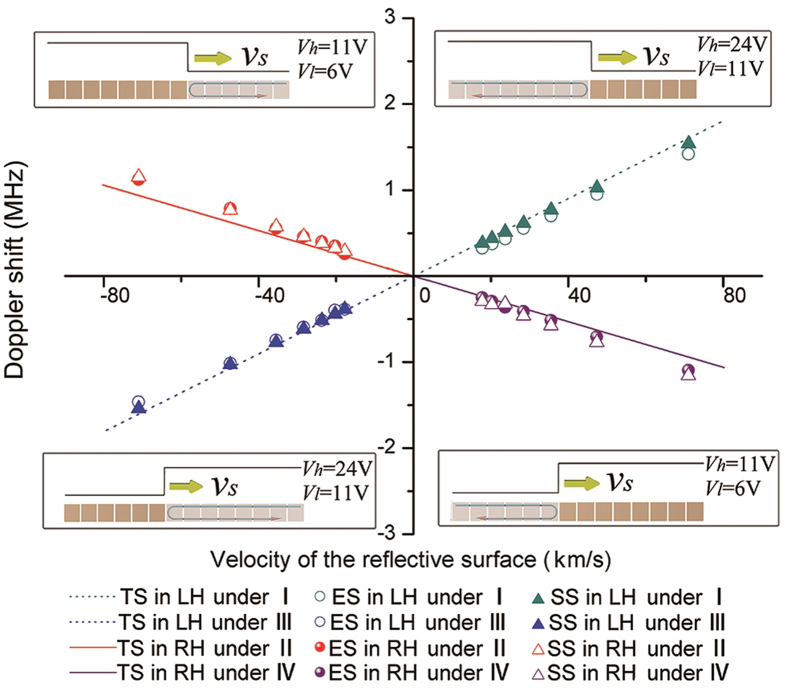
Theoretical, simulated and measured Doppler shifts under four different conditions indicated by I to IV. Four colors correspond to four different experimental conditions: I (*V*_*i*_ = 11 V,*V*_*h*_ = 24 V; the wave incident from the left); II (*V*_*i*_ = 6 V,*V*_*h*_ = 11 V; the wave incident from the right); III (*V*_*i*_ = 11 V,*V*_*h*_ = 24 V; the wave incident from the right); and IV (*V*_*i*_ = 6 V,*V*_*h*_ = 11 V; the wave incident from the left). (TS: theoretical shifts; ES: experimental shifts; SS: simulated shifts; LH: left-handed; RH: right-handed).

Figure 3 shows a comparison between the theoretical, simulated and experimental results of the Doppler frequency shifts in the different scenarios indicated as I to IV. In condition I (the left section of TL is set in high bias voltage *V*_*h*_ = 24 V(left-handed passband) while the right section of TL is set in low bias voltage *V*_*i*_ = 11 V(stop band); the wave is injected from the left end), the reflective boundary is moving away from the detector in the left-handed passband (*v*_*s*_ > 0). In condition II (the left section of TL is set in *V*_*h*_ = 11 V(stop band) while the right section of TL is set in *V*_*i*_ = 6 V(right-handed passband); the wave is injected from the right), the reflective boundary is approaching the detector in the right-handed passband (*v*_*s*_ < 0). In condition III (the left section of TL is set in *V*_*i*_ = 11 V (stop band) while the right section of TL is set in *V*_*h*_ = 24 V (left-handed passband); the wave is injected from the right), the reflective boundary is approaching the detector in the left-handed passband (*v*_*s*_ < 0). In condition IV (the left section of TL is set in *V*_*i*_ = 6 V (right-handed passband) while the right section of TL is set in *V*_*h*_ = 11 V (stop band); the wave is injected from the left), the reflective boundary is moving away from the detector in the right-handed passband (*v*_*s*_ > 0).

**Figure 4:**
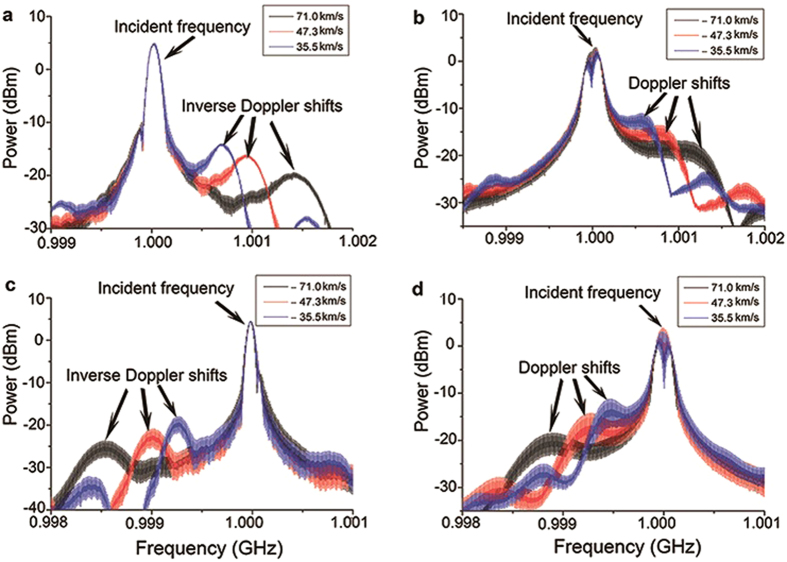
Experimental received spectra corresponding to the four conditions indicated by I to IV. **a**, **b**, **c**, **d** corresponding to the spectra under conditions I, II, III, IV separately.

Figure 4 only shows the measured frequency spectra at three different effective velocities of the reflective boundary, with a, b, c, d corresponding to the spectra under conditions I, II, III, IV separately. It can be seen that the inverse Doppler effect occurs under conditions I and III, while the normal Doppler effect occurs under conditions II and IV. The intensity of the incident frequency signal, which serves as a reference, is much stronger than those of the Doppler shifted signals in each of the four figures. The main reason for this is that the detector being connected to the returning end of the circulator, as shown in Fig. 1, picks up a strong reflection at the input of the transmission line, even though the 14 units of the transmission line are all set in passband (See in Supplementary Figure 9a). What’s more, it is difficult to establish a good impedance match between the connection of the circulator (50 Ω coaxial cable) and the tunable CRLH transmission line since the input impedance of the transmission line varies in the time domain with the change of the bias voltage. Therefore, the incident frequency component in Fig. 4 mostly accounts for the reflected wave at the input of the transmission line.

## Discussion

We have presented an unambiguous experimental realization of the inverse Doppler effect, as well as the normal Doppler effect, in an electronically reconfigurable RF metamaterial structure. The dispersion characteristic of the metamaterial, a CRLH transmission line unit, loaded with varactors, inductors and capacitors, can be tuned to provide a right-handed passband, band gap or left-handed passband by controlling the bias voltages. By this means an electronically controllable moving reflective boundary between the passband and band gap regions can be formed on the CRLH transmission line by the reflective boundary controller (a digital switching circuit). When an RF wave at 1 GHz is incident on an approaching (receding) reflective boundary formed on the CRLH transmission line, the boundary can be considered as an effective source of the reflected wave, moving towards (away from) the detector. The experiments demonstrate that the inverse Doppler effect can be observed when the CRLH transmission line is set in left-handed passband mode while the normal Doppler effect can be seen when the transmission line is set in right-handed passband mode. The maximum frequency shift is mainly limited by the performance of the components of the digital switching circuit, which lead to finite rise and decay times of the pulse signal (~20 ns for each) (see Supplementary Information S-I).

By applying a sequence of different bias voltages, the described electronically controlled composite right-left handed transmission line can easily realize the inverse Doppler shift over a frequency range between 0.5 GHz and 1.3 GHz. It is envisioned that this work has paved a way towards practical applications of the inverse Doppler effect in satellite navigation, radars and wireless communications^29^,^30^.

## Methods

### Fabrication of the 1D tunable composite right-left handed transmission line (CRLH TL)

The CRLH TL unit consists of five sections of microstrip lines with the same width *w* = 3 *mm* and different lengths (*d*_1_ = 2.5*mm*, *d*_2_ = 2*mm*), fabricated on FR-4 substrate (relative permittivity *ε*_*r*_ = 4.75) with thickness *h* = 1.6 mm, as shown in Supplementary Fig. 1. The distributed capacitance and inductance, and the characteristic impedance are: *C*_*R*_ = 1.2847×10^−10^F, *L*_*R*_ = 3.0847×10^−7^H and *Z*_0_ = 50Ω, respectively. The transmission line is loaded with three varactor diodes NXP BB135, two inductors *L*_*S*_ = 1.5 nH in series and one *L*_*p*_ = 6.3 nH in parallel. The three varactors work as three capacitors, two in series *C*_*s*_ and one in parallel *C*_*p*_, having the same capacitance under chosen bias voltages. The 4^th^ section of the CRLH TL unit shown in Fig. 1b can be considered to be divided into two identical parts with *C*_*p*_ inserted in the middle, indicated by the yellow sections in Supplementary Fig. 1. The lengths of each BB135 and of the inductors are all 0.8 mm. Hence the length of the unit cell of the CRLH TL is *d*_0_ = 14.2 mm. Supplementary Figure 2 is a photograph of the transmission line. The RF wave is emitted from one of the two end ports, while the other end port connects to a 50 Ω matching load. Bias voltages are applied from the branches. The different colors of the arrows near the ports indicate different conditions in states I to IV.

### Measurements of Doppler frequency shifts

An Agilent network analyzer N5222A was used to test the transmission characteristic of the composite right-left handed transmission line, which is given in Fig. 2c. All the units of the CRLH TL were supplied with the same bias voltage in this test. The set-up for observation of inverse Doppler shifts, shown in Fig. 1a, comprises an Agilent vector signal generator E8267D, a circulator with isolation 20 dB, an Agilent signal analyzer N9020A, the 1-D tunable CRLH TL, a 50Ω matching load, and the reflective boundary controller. The *Gate* function of the spectrum analyzer is used to select a certain time domain when the inverse Doppler shift occurs by using the voltage signal from the first unit of the reflective boundary controller to the analyzer as the trigger signal, and setting the duration of the inverse Doppler shift as the length of the *Gate* function, which is a function in the N9020A spectrum used to select the signal in a chosen time domain.

### Reflective boundary controller

The method of achieving a moving reflective boundary is to generate a sequence of bias voltage steps, in which the timing of the rising and falling edges is controllable. The circuit model of such a sequential bias voltage generator comprises a shift register, monostable multivibrators and amplifiers, shown in Supplementary Fig. 3, and explained in Supplementary Information S-I. The key feature of the circuit is the special function of the shift register, which is to move the input signal one unit rightwards at each falling edge of the clock^31^,^32^. This results in a discrete delay of the outputs, and the delay time of adjacent outputs is one clock period *τ*. Consequently, the rising or falling edge of the sequence of bias voltages is shifted *τ* rightward in the time domain (see Supplementary Information S-I and Supplementary Fig. 4). It is worth noting that, since the reflective boundary only moves when the bias voltages rise or fall, the inverse or normal Doppler effect occurs in the corresponding time domain. Thus the fast Fourier transform algorithm (FFT) should be used to show the frequency shift of the received signal in a chosen time domain. The average velocity of the reflective boundary is *v*_*s*_ = *d*_0_/*τ*, where *d*_0_ is the length of the transmission line unit. The velocity is positive when the boundary moves away from the source, and can be chosen by regulating the clock period *τ*.

### Simulation of the inverse and normal Doppler shifts

The CRLH TL unit shown in Supplementary Fig. 1 can be modeled by using the Agilent Advanced Design System (ADS) simulator. However, we modified the circuit model of the BB135, since the resonant frequency provided on the NXP website doesn’t match well with our measured results. Accordingly an inductance is placed in series with the original BB135 model as shown in Supplementary Fig. 7a. Fig. 7b and Fig. 7c are comparisons between the simulated and the experimental capacitances of the varactor and |*S*_21_| of a CRLH TL composed of 14 units as shown in Supplementary Fig. 2. The results match well under the chosen bias voltages at our working frequency of 1 GHz.

The square-wave voltage source component *Vtpulse* in ADS is applied to simulate the Doppler shifts on the CRLH TL. We can control the velocity of the reflective boundary by tuning the delay time and pulse width of the *Vtpulse*. Meanwhile, the function *fs* in ADS can help us to conduct an FFT of the signal in the time domain when the Doppler effect occurs. Supplementary Fig. 8 shows the simulated results.

## Additional Information

**How to cite this article**: Ran, J. *et al*. Realizing Tunable Inverse and Normal Doppler Shifts in Reconfigurable RF Metamaterials. *Sci. Rep*. **5**, 11659; doi: 10.1038/srep11659 (2015).

## Supplementary Material

Supplementary Information
