# Supplementary material for: Realizing Tunable Inverse and Normal Doppler Shifts in Reconfigurable RF Metamaterials
Source: Sci Rep. 2015 Jun 26;5:11659. doi: 10.1038/srep11659 (PMC4481520; doi:10.1038/srep11659)
Supplement: Supplementary Information [file srep11659-s1.pdf]

Supplementary Information for

Realizing Tunable Inverse and Normal Doppler Shifts in Reconfigurable RF Metamaterials

Jia Ran[1], Yewen Zhang[1*], Xiaodong Chen[2, 3*], Kai Fang[1], Junfei Zhao[1], Yong Sun[1], Hong Chen[1]

[1]*Tongji University, Shanghai, 200092, China*

[2]*University of Electronic Science and Technology of China, Chengdu, 610054, China*

[3]*Queen Mary University of London, E1 4NS, U.K.*

* Correspondence to yewen.zhang@tongji.edu.cn and xiaodong.chen@qmul.ac.uk

S-I : Fabrication of the reflective boundary controller

Supplementary Figure 3 is the circuit module of the reflective boundary controller. The serial-in/parallel-out (SIPO) shift register is a cascade of 14 edge-triggered JK flip-flops (FF: 74VHC112) connected in series, sharing one clock signal. As a form of sequential logic circuit, it can make the serial-in signal moved within the register from one flip-flop to another at each falling edge of the clock signal, resulting in a discrete delay of the parallel outputs, and the delayed time of the adjacent outputs is the clock's period τ , which is tunable. The rising edge of each short pulse outputted by the flip-flop would set the monostable multivibrator (MM: 74HC221A) entering a transient state, whose duration is determined by the resisters and capacitors in the circuit. Each of the 14 output signals is amplified by an amplifiers (Amp: LM7171BIM) before being provided to each unit of the CRLH TL as the bias voltages $V(x_i,t)$. The clock signal is a square wave with a period τ , while the trigger signal is a short pulse with a repetition rate of T , where $T >> \tau$. The trigger signal can trig the rising or the falling of the bias voltages from left to right on the transmission line, as shown in Supplementary Figure 4. .

The inverse or normal Doppler effect happens in the corresponding time domain $t \in \left[nT, nT+13\tau\right]$, where n is a non-negative integer. The position shift of the bias voltages is $x_i = d_0 \cdot i = d_0 \cdot \left\lfloor T\left\{t/T\right\}/\tau + 1\right\rfloor, i = 1,2...14$, where $\lfloor \ \rfloor$ is the floor function and $\{ \ \}$ is the fractional part function. The average velocity of the reflective boundary $v_s = d_0/\tau$, where d_0 is the length of the unit.

Supplementary Figure 4 is the schematic of the bias voltage profiles on the transmission line at different time under conditions indicated by II and IV. As we can

see that the rising edge is moving from the first unit to the 14th along with the time axis and the interval of each shift of the rising edge keeps the same with the period of the clock signal τ. The photograph of the reflective boundary controller is shown in Supplementary Fig. 5.

S-II : Theoretical calculation

For a two-port network having the transmission matrix $A = \begin{bmatrix} a & b \\ c & d \end{bmatrix}$, the inputs (voltage U_1, current I_1) and the outputs (voltage U_2, current I_2) have the following relationship according to the definition of the transmission matrix:

$$\begin{bmatrix} U_1 \\ I_1 \end{bmatrix} = \begin{bmatrix} a & b \\ c & d \end{bmatrix} \begin{bmatrix} U_2 \\ I_2 \end{bmatrix} \tag{1}$$

while

$$\begin{cases} U_2 = U_1 e^{-j\beta_{Bloch} d_0} \\ I_2 = I_1 e^{-j\beta_{Bloch} d_0} \end{cases} \tag{2}$$

where d_0 is the length of the network and β_{Bloch} is the propagation constant. Applying equation (2) to (1), we have:

$$\begin{bmatrix} 0 \\ 0 \end{bmatrix} = \begin{bmatrix} ae^{-j\beta_{Bloch} d_0} - 1 & be^{-j\beta_{Bloch} d_0} \\ ce^{-j\beta_{Bloch} d_0} & de^{-j\beta_{Bloch} d_0} - 1 \end{bmatrix} \begin{bmatrix} U_1 \\ I_1 \end{bmatrix} \tag{3}$$

In order to have nontrivial solution for U_1 and I_1, it has to have:

$$(ae^{-j\beta_{Bloch} d_0} - 1)(de^{-j\beta_{Bloch} d_0} - 1) - bce^{-2j\beta_{Bloch} d_0} = 0 \tag{4}$$

Since $\mathrm{ad} - bc = 1$ for a reciprocal network, the propagation constant can be obtained as:

$$|\beta_{Bloch}| = \frac{1}{d_0} \arccos \frac{a+d}{2} \tag{5}$$

The sign of β_{Bloch} depends on whether it is right-handed passband ($\beta_{Bloch} > 0$) or left-handed passband ($\beta_{Bloch} < 0$). The phase velocity of the wave is:

$$v = \frac{\omega}{\beta_{Bloch}} \tag{6}$$

The Doppler shift equations are:

$$\frac{f_r}{f_i} = \frac{1 - (\vec{v}_s \cdot \vec{v}_i)/v_i^2}{1 - (\vec{v}_s \cdot \vec{v}_r)/v_r^2} \tag{7}$$

$$\Delta f = f_r - f_i \tag{8}$$

where $f_r, f_i, \Delta f, v_s, v_i, v_r$ are the frequencies of the reflective wave and incident wave, the Doppler shift, and the velocity of the moving reflective boundary, incident wave phase velocity and reflective wave phase velocity, respectively.

The CRLH TL unit is a cascade network with 12 elements as shown in Supplementary Fig. 1.

1). The transmission matrices of the microstrip line sections in blue, purple, yellow in Supplementary Fig. 1 are:

$$A = \begin{bmatrix} \cos\theta_1 & jZ_0 \sin\theta_1 \\ \frac{j\sin\theta_1}{Z_0} & \cos\theta_1 \end{bmatrix}, B = \begin{bmatrix} \cos\theta_2 & jZ_0 \sin\theta_2 \\ \frac{j\sin\theta_2}{Z_0} & \cos\theta_2 \end{bmatrix}, C = \begin{bmatrix} \cos\theta_3 & jZ_0 \sin\theta_3 \\ \frac{j\sin\theta_3}{Z_0} & \cos\theta_3 \end{bmatrix} \tag{9}$$

where $\theta_1 = \beta d_1, \theta_2 = \beta d_2, \theta_3 = \frac{\beta d_2}{2}$, and $\beta = 2\pi f \sqrt{L_R C_R}$, f is the frequency of the incident wave and here $f = 1$ GHz.

2). The transmission matrices of the inductance in parallel and in series are:

$$D = \begin{bmatrix} 1 & 0 \\ \frac{1}{j\omega L_p} & 1 \end{bmatrix}, E = \begin{bmatrix} 1 & j\omega L_s \\ 0 & 1 \end{bmatrix} \tag{10}$$

3). The transmission matrices of the capacitor in parallel and in series are:

$$F=\begin{bmatrix} 1 & 0 \\ j\omega C & 1 \end{bmatrix}, G=\begin{bmatrix} 1 & \frac{1}{j\omega C} \\ 0 & 1 \end{bmatrix} \tag{11}$$

Since these 12 sections are cascade connected, we can obtain the final transmission matrix of this unit:

$$H = ADAGBECFCEBG \tag{12}$$

According to equations (5) and (6), we can obtain the phase velocity of the incident wave,

$$|v| = \frac{\omega}{\frac{1}{d_0}\arccos\frac{h_{11}+h_{22}}{2}} \tag{13}$$

where h_{ij} is the element in row *i* and column *j* of transmission matrix H.

We can obtain the algebraic solution of the phase velocity and plot it in Supplementary Fig. 6. It illustrates that the dispersion of the CRLH TL is linear in the measured Doppler shift rang (± 1.5 MHz) and the velocity varies slightly in this range. Hence we have approximately $\vec{v}_i \approx -\vec{v}_r$, which simplifies equation (7) to :

$$\frac{f_r}{f_i} = \frac{v_i^2 - \vec{v}_s \cdot \vec{v}_i}{v_i^2 + \vec{v}_s \cdot \vec{v}_i} \tag{14}$$

S-III : Properties of the moving reflecting boundary and the Doppler shift calculation

The reflection coefficient of the reflecting boundary is varying within the variation of the number of the units set in band gap. The strongest reflection happens when the 14 units are all set in band gap. The reflection coefficient is 0.76 at this case. While the transmission coefficient is 0.003. Hence there is a lot of loss in the units set in band gap. This loss is mainly due to dielectric loss in the medium and the Ohmic dissipation in the varactors. The Supplementary Figure 9b is the theoretical effective relative permittivity and permeability of the transmission line set in band gap, showing that the band gap is a magnetic negative gap.

Supplementary Figure 10 has proposed a way to predict and explain the Doppler shifts. For each experimental condition we measure the velocity of the moving reflective boundary, and draw the Doppler shift line across the dispersion line at 1 GHz. The intersection of the Doppler shift line and the dispersion line is the frequency of the reflected wave and the $\pm\delta f$ are the Doppler shifts. Supplementary Fig. 10a is under the condition that the reflective boundary is moving away from the detector, hence the slope of the Doppler shift line is positive. The frequency is up-shifted on the red line, which represents the left-handed passband and the frequency is down-shifted on the blue line that indicates the right-handed passband, as the inverse and normal Doppler effects suggest in the first and fourth quadrants in Figure 4. Similarly, Supplementary Fig. 10b tells the Doppler shifts when the reflective boundary is approaching to the detector, corresponding to the second and third quadrants in Figure 4.

SUPPLEMENTARY FIURES

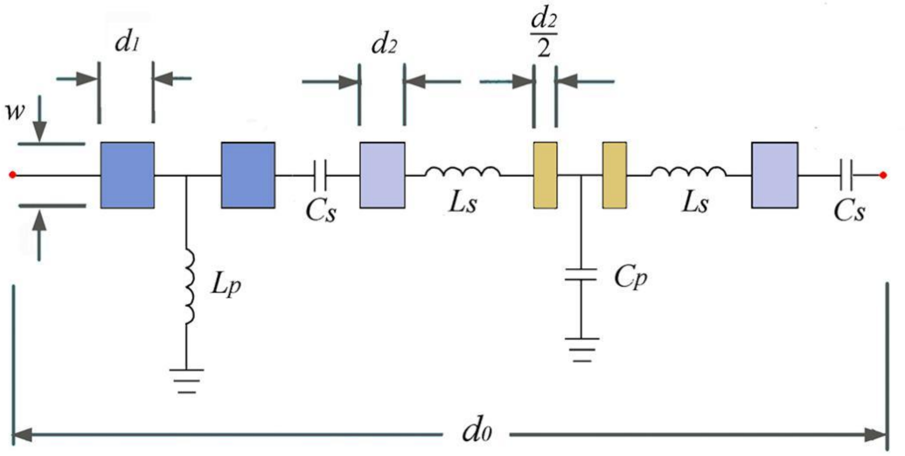

Supplementary Figure 1 | The equivalent circuit diagram of the transmission line's unit.

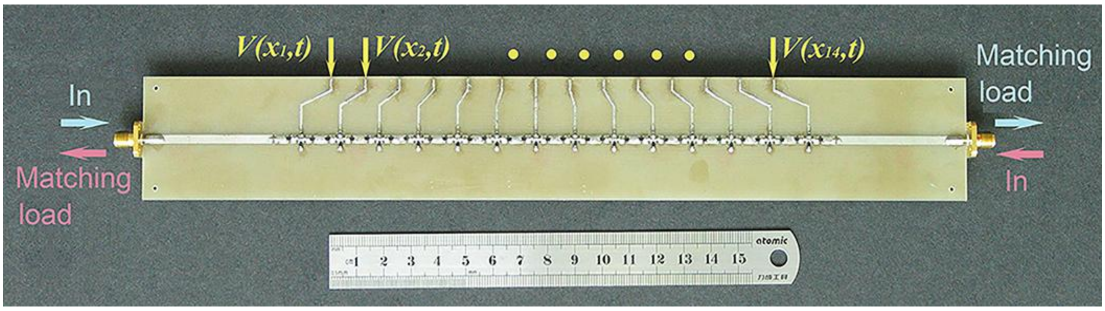

Supplementary Figure 2 | Photograph of the CRLH TL. The blue arrows are under conditions indicated by Ⅰ, Ⅳ while the red arrows are under conditions indicated by Ⅱ, Ⅲ.

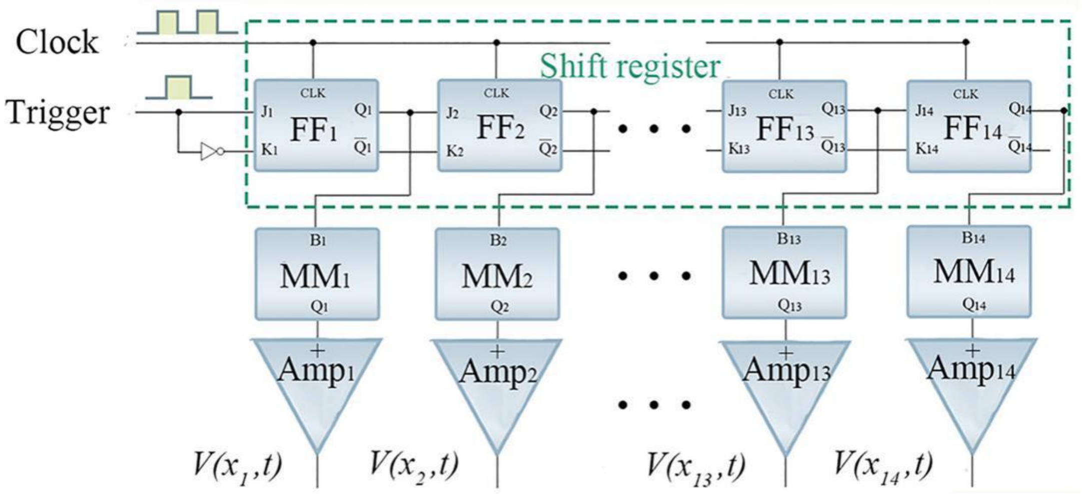

Supplementary Figure 3 | Module of the reflective boundary controller. The clock and trigger signals are square waves whose periods are tunable. Shift register is combined by 14 edge-triggered JK flip-flops 74VHC112, while MM_i $(i=1,2...14)$ are monostable multivibrators 74HC221A. Amp_i $(i=1,2...14)$ are amplifiers LM7171BIM. $V(x_i,t)$ $(i=1,2...14)$ are the parallel voltage outputs whose rising (falling) edges are moving from left to right as shown in Supplementary Fig. 4.

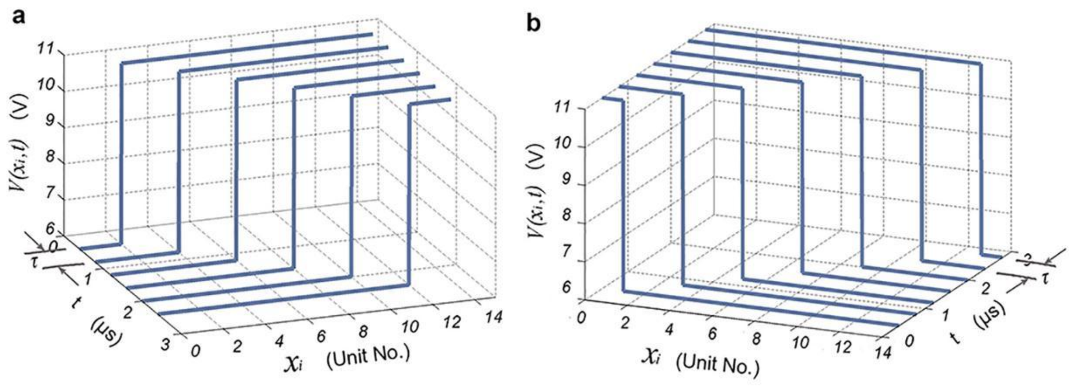

Supplementary Figure 4 | Schematic of the bias voltages of each transmission line unit dependent of time. a, b are the sets of bias voltages chosen to realize the normal Doppler effect under condition Ⅳ and Ⅱ indicated in Fig. 3 respectively. The unit of x_i is the ordinarily number of the units of the transmission line. τ is the period of the clock signal.

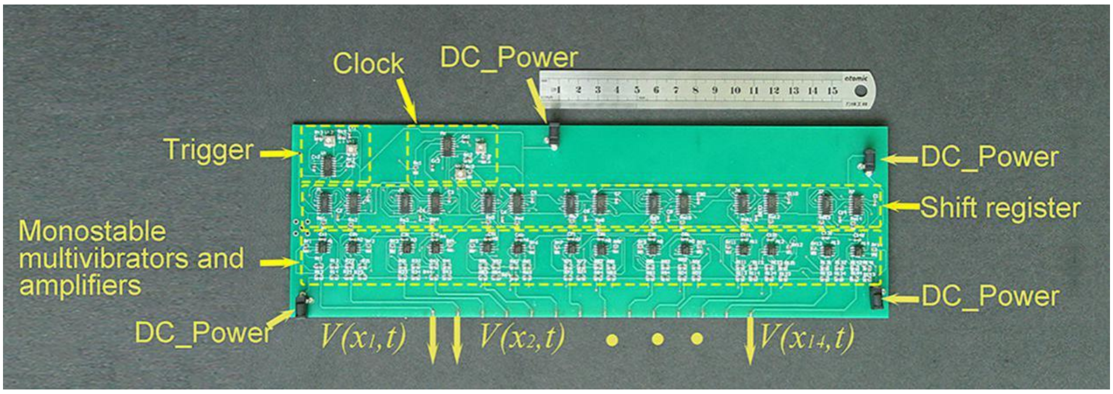

Supplementary Figure 5 | Photograph of the reflective boundary controller. It is mainly constituted by four parts: trigger module, clock module, shift register module, monostable multivibrators and amplifiers.

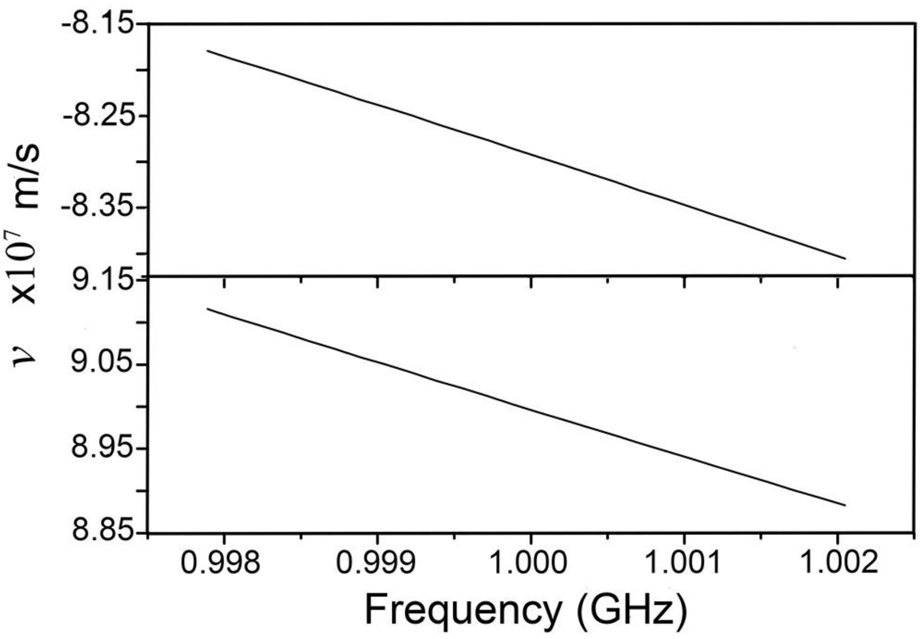

Supplementary Figure 6 | The theoretical velocity of the wave include the measured Doppler shift rang (± 1.5 MHz). v is the phase velocity of the wave. The figure above is the phase velocity of wave propagating in left-handed passband transmission line, while the figure blow is the phase velocity of wave propagating in right-handed passband.

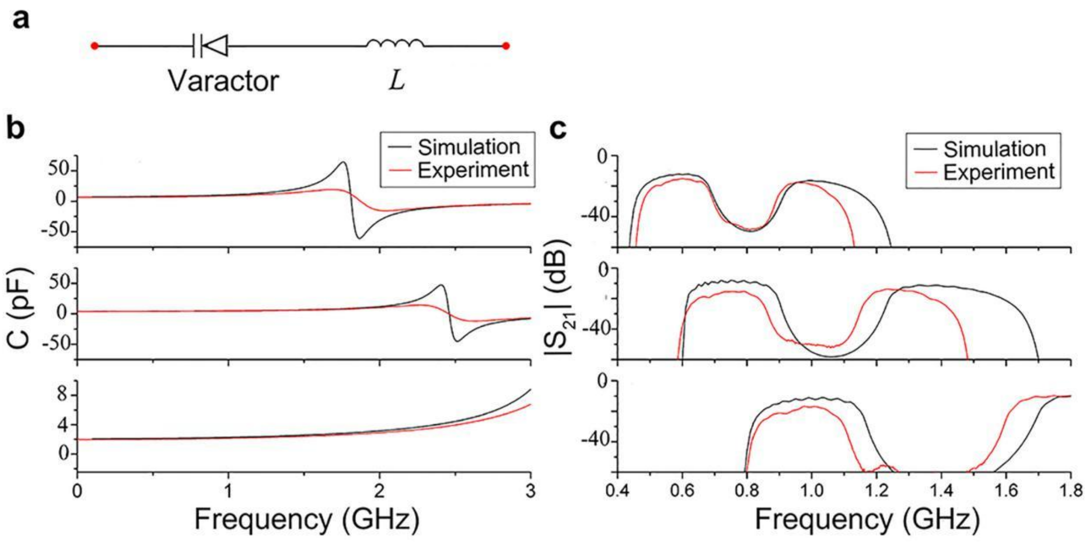

Supplementary Figure 7 | Schematic and performance of the improved model of varactor BB135 in Agilent Advanced Design System (ADS). a, Schematic of the improved model of varactor Philips BB135. The varactor is the original model downloaded from the Philips website. *L* is inductance of 1.041 nH. b, Comparison between the simulated and experimental capacitances dependent on frequency under three bias voltages *V* which are chosen to realize the normal and inverse Doppler Effect. The bias voltages in simulation are 5 V, 11 V, 23 V seen from above, while the bias voltages in experiment are 6 V, 11 V, 24 V. c, Comparison between the simulated and experimental $|S_{21}|$ under three bias voltages *V* chosen to realized the normal and inverse Doppler Effect. The bias voltages in simulation and experiment are as same as b.

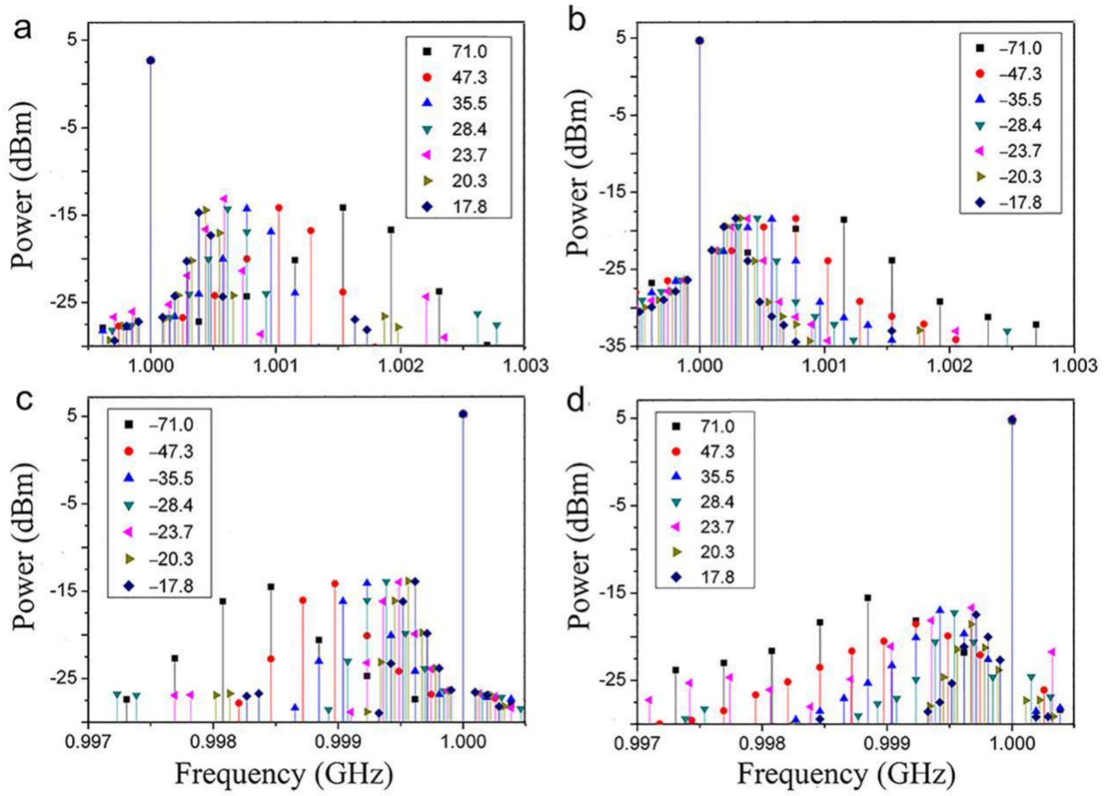

Supplementary Figure 8 | Simulated spectrums of Doppler shifts happened at seven velocities of the reflective boundary (unit km/s). a, b, c, d are corresponding to conditions Ⅰ, Ⅱ, Ⅲ, Ⅳ indicated in Fig. 3. Those noise waves are induced by the discontinuity of the moving of the reflective boundary.

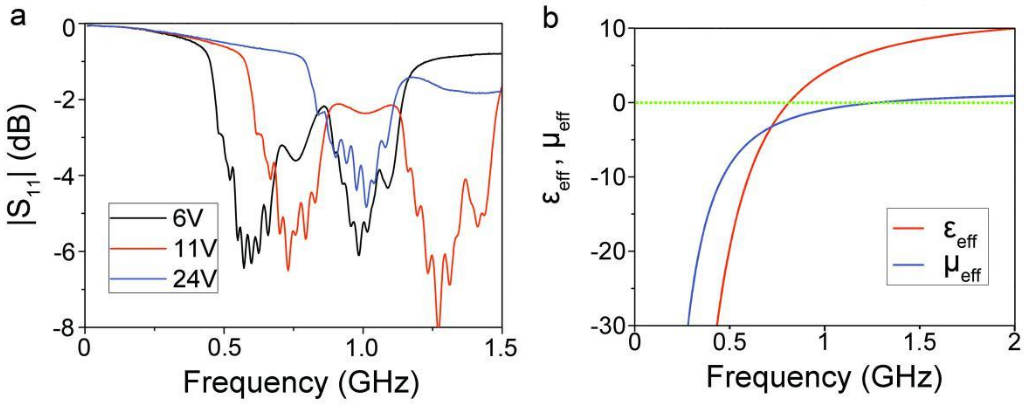

Supplementary Figure 9 | Properties of the reflecting surface. **a**, |S11| of the transmission line set in 6 V, 11 V, 24 V separately. **b**, Theoretical effective permittivity and permeability of the transmission line set in band gap. The dotted green line indicates 0 value.

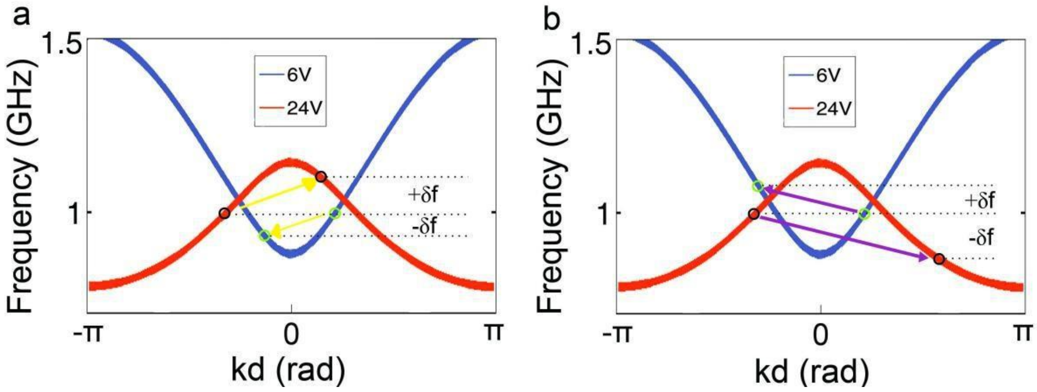

Supplementary Figure 10 | The Doppler shift line imposed on dispersion lines.

The red lines are dispersion lines under 24 V and 1 GHz is set in left-handed passband. While the blue ones are those under 6 V and 1 GHz is set in right-handed passband. Yellow and purple lines are the Doppler shift lines **a**, the reflective boundary is moving away from the source that the slope of the Doppler shift lines is positive. **b**, the reflective boundary is approaching the source that the slope of the Doppler shift is negative.
